# Evolutionary History of the Vertebrate Mitogen Activated Protein Kinases Family

**DOI:** 10.1371/journal.pone.0026999

**Published:** 2011-10-26

**Authors:** Meng Li, Jun Liu, Chiyu Zhang

**Affiliations:** Institute of Life Sciences, Jiangsu University, Zhenjiang, Jiangsu, China; University of Texas, United States of America

## Abstract

**Background:**

The mitogen activated protein kinases (MAPK) family pathway is implicated in diverse cellular processes and pathways essential to most organisms. Its evolution is conserved throughout the eukaryotic kingdoms. However, the detailed evolutionary history of the vertebrate MAPK family is largely unclear.

**Methodology/Principal Findings:**

The MAPK family members were collected from literatures or by searching the genomes of several vertebrates and invertebrates with the known MAPK sequences as queries. We found that vertebrates had significantly more MAPK family members than invertebrates, and the vertebrate MAPK family originated from 3 progenitors, suggesting that a burst of gene duplication events had occurred after the divergence of vertebrates from invertebrates. Conservation of evolutionary synteny was observed in the vertebrate MAPK subfamilies 4, 6, 7, and 11 to 14. Based on synteny and phylogenetic relationships, MAPK12 appeared to have arisen from a tandem duplication of MAPK11 and the MAPK13-MAPK14 gene unit was from a segmental duplication of the MAPK11-MAPK12 gene unit. Adaptive evolution analyses reveal that purifying selection drove the evolution of MAPK family, implying strong functional constraints of MAPK genes. Intriguingly, however, intron losses were specifically observed in the MAPK4 and MAPK7 genes, but not in their flanking genes, during the evolution from teleosts to amphibians and mammals. The specific occurrence of intron losses in the MAPK4 and MAPK7 subfamilies might be associated with adaptive evolution of the vertebrates by enhancing the gene expression level of both MAPK genes.

**Conclusions/Significance:**

These results provide valuable insight into the evolutionary history of the vertebrate MAPK family.

## Introduction

The mitogen activated protein kinase (MAPK) cascade consists of three protein kinases, MAPK, MAPK kinase (MAPKK) and MAPKK kinase (MAPKKK) [1]. In the classical three-tiered MAPKKK–MAPKK–MAPK cascade, MAPKKKs phosphorylate and activate specific MAPKKs, which further phosphorylate and activate downstream MAPKs [2]. All eukaryotic cells use multiple MAP kinase modules for signal transduction and the MAPK pathways are involved in diverse cellular processes, including cell growth [3], [4], migration [5], survival [6], mesoderm differentiation [7], spermatozoa maturation [8], and other essential functions in development [9], [10], [11], [12], [13], [14], [15]. Recently, there is an increasing understanding of roles that MAPKs play in diseases [16], [17], [18], [19]. MAPKs are involved in the resistance to tamoxifen, and MAPK-specific phosphatases are associated with resistance to treatment with doxorubicin, mechlorethamine, paclitaxel and proteasome inhibitors, and oxidative-stress-induced cell death in breast cancer [20]. In addition, P38 MAPKs participate in some events related to Alzheimer's disease (e.g. excitotoxicity, synaptic plasticity and tau phosphorylation) [21], suggesting that they may become new Alzheimer's disease treatment strategies [22].

The MAPK family is conserved in plants, fungi and animals [2], [16]. Since the identification of the first member of a MAPK family in the 1980s [23], a great deal of MAPKs have been reported. Six different MAPK cascades have been characterized in mammals including extracellular signal-regulated kinases (ERK)1/2 (also known as MAPKs 1&3), ERK3/ERK4 (MAPKs 6&4), ERK5 (MAPK7), ERK7/8 (MAPK15), JNK (c-Jun N-terminal kinases, also known as MAPKs 8–10) and P38 (MAPKs 11–14) [10], [11], [24], [25]. In addition, according to the ability to be phosphorylated and activated by MAPK kinases, the MAPKs were further classified into conventional and atypical enzymes [26]. The former including ERK1/ERK2, JNK, P38 [27], and ERK5 (MAPK7) can be phosphorylated and activated by the MAPKKs, whereas the latter that include ERK3/ERK4 and ERK7/8 can not.

The MAPK signal transduction pathway plays a pivotal role in eukaryotic cellular regulation. A previous study indicated that MAPKs might originate from an ancestral ERK before the separation between animal, fungal, and plant lineages [28]. However, because only limited sequences and species from vertebrates were used, it did not provide a full framework to all the vertebrate MAPKs in detail. The evolutionary history of the vertebrate MAPK family needs to be confirmed by systematic phylogenetic analyses. Furthermore, whether natural selection has driven the evolution of the vertebrate MAPK family remains unknown. In this study, we investigated the phylogenetic and molecular evolution of the vertebrate MAPK family more thoroughly. We found that the vertebrate MAPK family might have originated from 3 earlier progenitors and experienced an expansion through gene duplications during the early evolution of the vertebrates, in which conserved gene synteny were observed. The intron losses were specifically found in the MAPK subfamilies 4 and 7 during the vertebrate evolution from teleosts to amphibians and mammals. Purifying selection and conserved motifs were also detected in the MAPK subfamilies.

## Results

### MAPK family might experience an expansion after the divergence of vertebrates from invertebrates

In order to better understand the evolutionary history of the MAPK family in vertebrates, we generated a primary amino acid sequence data set covering mammals, birds, reptiles, amphibians and teleosts. To investigate the possible origin of vertebrate MAPKs, we extended this data set to include MAPKs from *Ciona intestinalis, Ciona savignyi* and *Strongylocentrotus purpuratus*. The phylogeny of the species involved in this study is shown in Figure S1.

The numbers of MAPK genes in these species are listed in Table 1. The NLK (Nemo-like kinase) family was not included in Table 1 as it has not been clearly classified into MAPKs. Except for a few species, most vertebrates possessed 8–13 MAPK family members. In *Danio rerio*, we identified 11 MAPKs, more than the 10 MAPKs having been reported previously [29]. However, in some vertebrate species (i.e. *Ovis aries*, *Sus scrofa*, *Tursiops truncatus*, and *Ornithorhynchus anatinus*), less than or equal to 6 MAPKs were identified, possibly due to incomplete or low quality genome sequencing. In tunicates *Ciona intestinalis*, *Ciona savignyi* and echinoderm *Strongylocentrotus purpuratus*, 5, 2 and 2 MAPKs were found, respectively.

**Table 1:** The total number of MAPK proteins found in the genomes of selected species.

Species	Number of MAPKs	Species	Number of MAPKs
*Homo sapiens*	13	*Gallus gallus*	9
*Macaca mulatta*	8	*Taeniopygia guttata*	9
*Pan troglodytes*	9	*Gasterosteus aculeatus*	11
*Mus musculus*	13	*Oryzias latipes*	11
*Rattus norvegicus*	12	*Takifugu rubripes*	9
*Ornithorhynchus anatinus*	6	*Tetraodon nigroviridis*	10
*Monodelphis domestica*	10	*Anolis carolinensis*	10
*Bos taurus*	13	*Xenopus tropicalis*	11
*Canis lupus familiaris*	13	*Tursiops truncatus*	4
*Equus caballus*	11	*Ciona intestinalis* *	5
*Ovis aries*	2	*Ciona savignyi* *	2
*Sus scrofa*	6	*Strongylocentrotus purpuratus* *	2
*Danio rerio*	11		

Asterisks indicate invertebrates.

From the numbers of the MAPK family members, it is obvious that the invertebrate species had less MAPKs than the vertebrate species. In addition, except for several MAPK6 pseudogenes observed in human and mouse [30], all the vertebrates had exactly one representative ortholog to each MAPK family member. These results suggest that the MAPK gene family might experience an expansion after the divergence of vertebrates from invertebrates.

### Phylogenetic analyses of the vertebrate MAPKs

**Figure 1:**
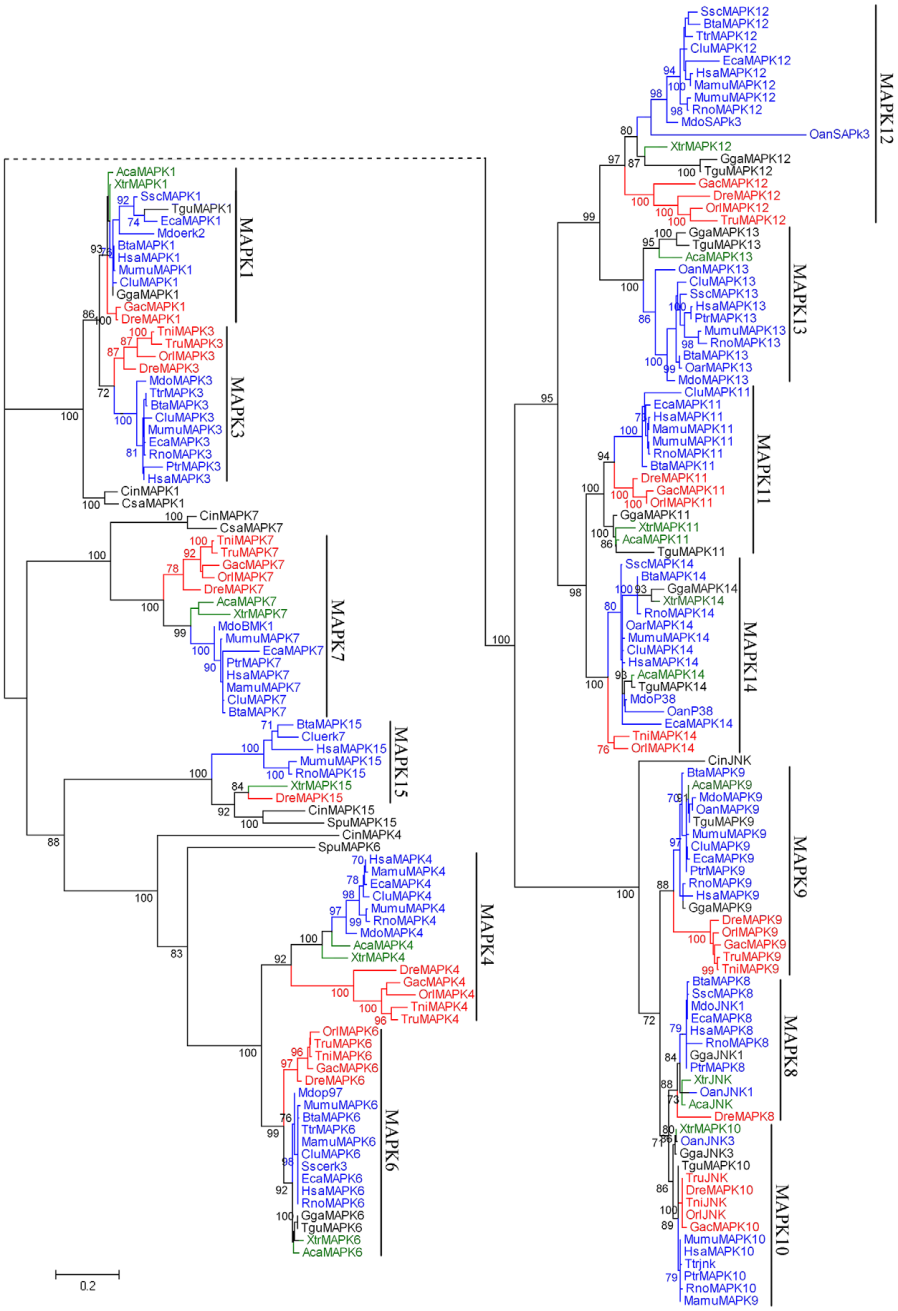
Maximum likelihood (ML) phylogenetic tree of the vertebrate MAPK family. The ML tree was constructed based on the protein sequences of the MAPK family using PHYML v2.4 with 100 bootstrap replications. The tree is unrooted and only the bootstrap values >70% are shown at interior nodes. The MAPK protein sequences from mammals, amphibians/reptiles and teleosts are marked in blue, green and red, respectively. The scale bar indicates the branch length that corresponds to 0.2 substitutions per site. The species and accession numbers are listed in Table S1. The corresponding amino acid sequence alignment is provided in Figure S2. The abbreviations used are as follows: Hsa, *Homo sapiens*; Mamu, *Macaca mulatta*; Ptr, *Pan troglodytes*; Mumu, *Mus musculus*; Rno, *Rattus norvegicus*; Oan, *Ornithorhynchus anatinus*; Mdo, *Monodelphis domestica*; Bta, *Bos taurus*; Clu, *Canis lupus familiaris*; Eca, *Equus caballus*; Oar, *Ovis aries*; Ssc, *Sus scrofa*; Dre, *Danio rerio*; Gga, *Gallus gallus*; Tgu, *Taeniopygia guttata*; Gac, *Gasterosteus aculeatus*; Orl, *Oryzias latipes*; Tru, *Takifugu rubripes*; Tni, *Tetraodon nigroviridis*; Aca, *Anolis carolinensis*; Xtr, *Xenopus tropicalis*; Ttr, *Tursiops truncatus*; Cin, *Ciona intestinalis;* Csa, *Ciona savignyi;* Spu, *Strongylocentrotus purpuratus.*

A phylogenetic tree was constructed with the conserved protein kinase regions from all complete vertebrate MAPK proteins (Figure 1). From the tree, we noted that all the genes from different vertebrate species within each MAPK subfamily clustered together to form an independent group, indicating that differentiation of the MAPK subfamilies occurred by gene duplications prior to the species separation of the vertebrates. In addition, except for the MAPK subfamily 7, either two or three of the other MAPK subfamilies clustered closely together to form a large group (Figure 1), indicating that they might share common ancestors and have originated through relatively recent gene duplications. In each large group, at least one subfamily contained all the three subgroups of teleosts, amphibians and mammals (Figure 1). Interestingly, the MAPK subfamilies 3, 9 and 13 were not found in amphibians compared with their sister subfamilies (1, 8 and 10, and 11, respectively). Similarly, the MAPK subfamily 13 was not found in teleosts compared with its sister subfamily 12 (Figure 1). These suggest that evolutionary loss of some MAPK subfamilies might have occurred in amphibians or teleosts after gene duplications.

It is apparent that the teleost subgroups, consisting of *Tetraodon nigroviridis, Gasterosteus aculeatus, Oryzias latipes, Takifugu rubripes* and *Danio rerio*, were always isolated from other vertebrates by each MAPK subfamily (Figure 1). Similarly, the amphibians/reptiles and the mammals subgroups were isolated from each other by most MAPK subfamilies. In the MAPK subfamilies 1, 4, 6, 7 and 12, the teleost subgroup was located at the root position, the amphibian subgroup at middle position, and the mammalian subgroup at the interior position. The relationships of the three subgroups were consistent with the known species phylogeny from aquatic vertebrates to semiaquatic and terrestrial vertebrates (Figure S1).

### Evolutionary origins of the vertebrate MAPK subfamilies

**Figure 2:**
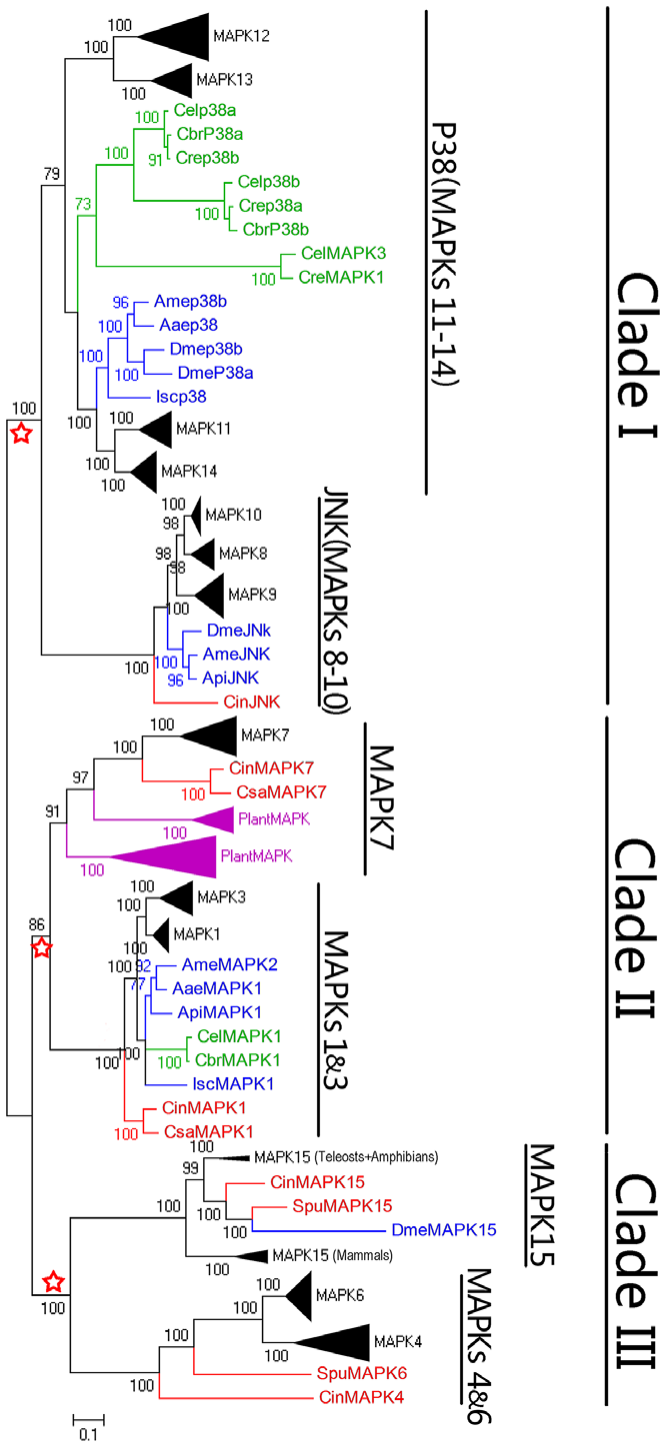
Bayesian phylogenetic tree of the MAPK protein sequences from vertebrates, tunicates, echinoderm, nematodes, arthropods and plants. The red stars indicate 3 earlier progenitors of the MAPK family. The tree is unrooted and Bayesian posterior probability values (>70%) are shown at interior nodes. The scale bar corresponds to 0.1 substitutions per site. The branches that correspond to vertebrates, echinoderm and tunicates, nematodes, arthropods, and plants are marked in black, red, green, blue, and purple, respectively. The species and accession numbers are listed in Table S1. The corresponding amino acid sequence alignment is provided in Figure S3. The abbreviations used are as follows: Isc, *Ixodes scapularis;* Aae, *Aedes aegypti;* Ame, *Apis mellifera;* Dme, *Drosophila melanogaster;* Api, *Acyrthosiphon pisum;* Cbr, *Caenorhabditis brenneri;* Cre, *Caenorhabditis remanei;* Cel, *Caenorhabditis elegans.*

To investigate the evolutionary origin of the MAPK subfamilies, we searched for the available MAPK orthologs in some invertebrates. The finding of orthologs in the genomes of nematodes (*Caenorhabditis briggsae, Caenorhabditis remanei* and *Caenorhabditis elegans*) and arthropods (*Apis mellifera, Drosophila melanogaster, Ixodes scapularis, Aedes aegypti and Acyrthosiphon pisum*), suggests that the MAPK family is conserved across invertebrates and vertebrates, and most members of the MAPK family have been established prior to the diversification of invertebrates and vertebrates. Gene duplications were found to be ubiquitous in the evolutionary history of the vertebrate MAPKs, such as MAPKs 1&3, MAPKs 4&6, MAPKs 8–10 and MAPKs 11–14 (Figure 2). In the P38 (MAPKs 11–14) subclade, MAPK genes from arthropods and nematodes formed two independent subgroups, and gene duplications were also observed. Interestingly, gene duplications might have occurred prior to the species separation of nematodes but posterior to the species separation of arthropods (Figure 2). In the JNK (MAPKs 8–10) subclade, both the MAPK subfamilies 8 and 10 clustered together and then clustered with the MAPK subfamily 9. The MAPK gene (CinJNK) from *Ciona intestinalis* was located at the root position of the MAPKs 8–10 subclade, indicating that a CinJNK-like gene might be the common ancestor of the vertebrate MAPKs 8–10. In addition, the JNK (MAPKs 8–10) and P38 (MAPKs 11–14) subclades formed one large clade I (bootstrap value: 100) (Figure 2), implying that the MAPK subfamilies 8 to 14 had originated from an earlier common progenitor.

In the MAPKs 1&3 subclade, the vertebrate MAPK subfamilies 1 and 3 clustered together, and the genes from arthropods and nematodes clustered together (Figure 2). The MAPKs of the marine invertebrates clustered outermost. No gene duplication was found in these invertebrate MAPK genes. For MAPK7 subfamily, two orthologs from marine invertebrates and one from nematodes were found. The vertebrate MAPK7 genes clustered together as an independent branch adjacent to the plant MAPK family. The MAPKs 1&3 and MAPK 7 subclades formed another large clade II (bootstrap value: 86) (Figure 2), implying that MAPK subfamilies 1, 3 and 7 have an earlier common progenitor.

In the MAPKs 4&6 subclade, the vertebrate MAPK subfamilies 4 and 6 clustered together and then clustered with the genes from marine invertebrates (Figure 2). *Ciona intestinalis* had one clear ortholog for the MAPK4 or MAPK6, arguing against that the MAPK4 and MAPK6 genes were restricted to chordates or vertebrates [26]. Thus the origin of MAPK4 and MAPK6 should predate the emergence of the common ancestor of echinoderms and chordates, about 550 million years ago [31]. The orthologs of MAPK15 were also identified in the genomes of *Drosophila melanogaster*, *Ciona intestinalis and Strongylocentrotus purpuratus*. These genes divided the vertebrate MAPK15 genes into two branches. The mammalian MAPK15 genes characterized one branch, and the amphibian and teleost MAPK15 were characteristic of the other (Figure 2). The MAPKs 4&6 and MAPK15 subclades formed the third large clade III (bootstrap value: 100) (Figure 2), suggesting that the vertebrate MAPK subfamilies 4, 6 and 15 had been derived from the third earlier common progenitor (Figures 1 and 2). Taken together, the vertebrate MAPK family might have originally originated from 3 earlier progenitors.

### Conservation of synteny in MAPK genes during the vertebrate evolution

**Figure 3:**
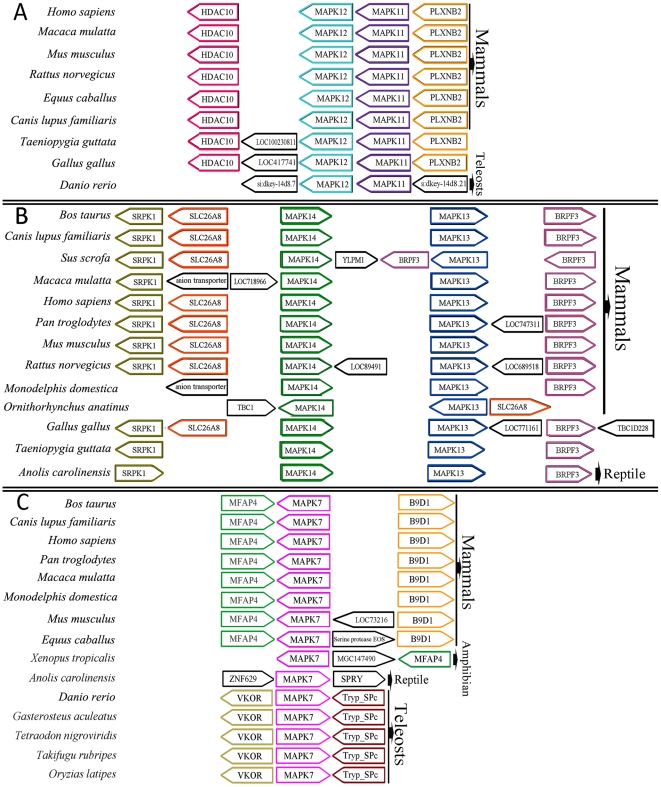
Order and orientation of genes syntenic to (A): MAPK11 and MAPK12, (B): MAPK13 and MAPK14, and (C): MAPK7. Genes are intentionally aligned in columns to facilitate visualization of synteny. For other details, please see text.

Orthologous relationships between the MAPK family members can be confirmed with conserved syntenies that appear to be particularly prevalent in the vertebrate MAPK family (Figures 3 and S4). In all the genomes studied to date, both MAPK11 and MAPK12 were present as tandem duplications in one chromosome and their local gene orders were very preserved (Figure 3A). In all the mammals analyzed here, the gene unit of MAPK11-MAPK12 was flanked by HDAC10 at left and PLXNB2 at right, showing an obvious syntenic relationship. In *Gallus gallus* and *Taeniopygia guttata*, LOC417741 and LOC100230811 lay between HDAC10 and MAPK12. However, in *Danio rerio*, the MAPK11-MAPK12 unit had different flanking genes from those in mammals and birds.

Like MAPK11 and MAPK12, both MAPK13 and MAPK14 were also present as tandem duplications in one chromosome (Figure 3B). Their local gene orders were preserved in the majority of the vertebrate species. Generally, MAPK14 and MAPK13 were adjacent to each other in the chromosome, with SRPK1/SLC26A8 flanking MAPK14 at left and BRPF3 flanking MAPK13 at right. This structure was relatively conserved in the mammalian genomes with some exceptions. For instance, several genes were inserted between SRPK1 and MAPK14 in *Macaca mulatta*, between MAPK14 and MAPK13 in *Rattus norvegicus* and *Sus scrofa* or between MAPK13 and BRPF3 in *Rattus norvegicus* and *Pan troglodytes*. These inserted genes might be attributed to relatively recent insertion events, showing different patterns of genomic evolution between species. Intriguingly, in *Sus scrofa*, MAPK13 was flanked by two BRPF3 genes and the structure unit of BRPF3-MAPK13-BRPF3 had a reverse gene orientation compared with most other species. The unique structure unit may be formed by a gene duplication event and a chromosome inversion. In this process, the downstream gene (BRPF3) of MAPK13 was duplicated and then inserted into its upstream. In addition, BRPF3 and SRPK1/SLC26A8 were also observed to be contiguous to MAPK13 and MAPK14 in the *Gallus gallus* genome. However, the SRPK1 and both BRPF3 and SRPK1 were absent in *Monodelphis domestica* and *Ornithorhynchus anatinus*, respectively.

MAPK11 & MAPK14 and MAPK12 & MAPK13 were two pairs of duplicated sister genes according to the phylogeny (Figures 1 and 2). The tight co-locations of MAPK11 and MAPK12 in one chromosome, as well as MAPK13 and MAPK14 in another one indicate that the two pairs of duplicated sister genes most likely arose from one segmental duplication event (Figure 3, A and B). Because duplicated genes are generally assumed to be functionally redundant at the time of origin, the usual fate awaiting most duplicated genes is silencing rather than preservation [32]. The fact that the MAPK11-MAPK12 unit was more conserved than the MAPK13-MAPK14 unit in gene synteny (Figure 3, A and B), together with the observation that the MAPK11-MAPK12 unit was identified in three species groups of mammals, teleosts and amphibians, whereas MAPK13 was not identified in teleosts (Figure 1), suggests that the latter might be the duplicated copy of the former during the segmental duplication. Compared with MAPK12, MAPK11 had closer genetic relationship with the ancestral MAPK genes of invertebrates, implying that MAPK11 arose earlier than MAPK12 in evolutionary history, that is, MAPK12 was the duplicated copy of MAPK11. Another compelling evidence in support of this conclusion was the observation that one or more genes (or locus) was often inserted into between MAPK13 and MAPK 14, whereas no gene (or locus) was inserted into between MAPK11 and MAPK 12 (Figure 3, A and B). A case in point was that in rat there was one locus (LOC89491) appearing between MAPK13 and MAPK14 genes, but none between MAPK11 and MAPK12 genes. Taken together, these results suggest that the MAPK subfamilies 11–14 might have arisen from a tandem duplication followed by a segmental duplication.

Similar syntenic relationships were found in the MAPK subfamilies 4, 6, and 7 (Figures 3C and S4). Intriguingly, the flanking genes of MAPK7 were obviously different between mammals and teleosts (Figure 3C). The gene order in mammals was often MFAP4 followed by MAPK7 and then B9D1, except for an absence of MFAP4 in *Macaca mulatta* and an additional inserted gene between MAPK7 and B9D1 in *Mus musculus* or *Equus caballus*. Distinct from those in mammals, the teleost MAPK7 genes were flanked by Tryp_SPc and VKOR, showing highly conserved gene order. In *Xenopus tropicalis*, MFAP4 was located at downstream of MAPK7 and had a different gene orientation from that of mammals. The unique syntenic evolution of MAPK7 might suggest that MAPK7 was associated with the vertebrate evolution from teleosts to amphibians and mammals compared with other MAPKs.

### Gene structure analyses of the vertebrate MAPK genes

**Figure 4:**
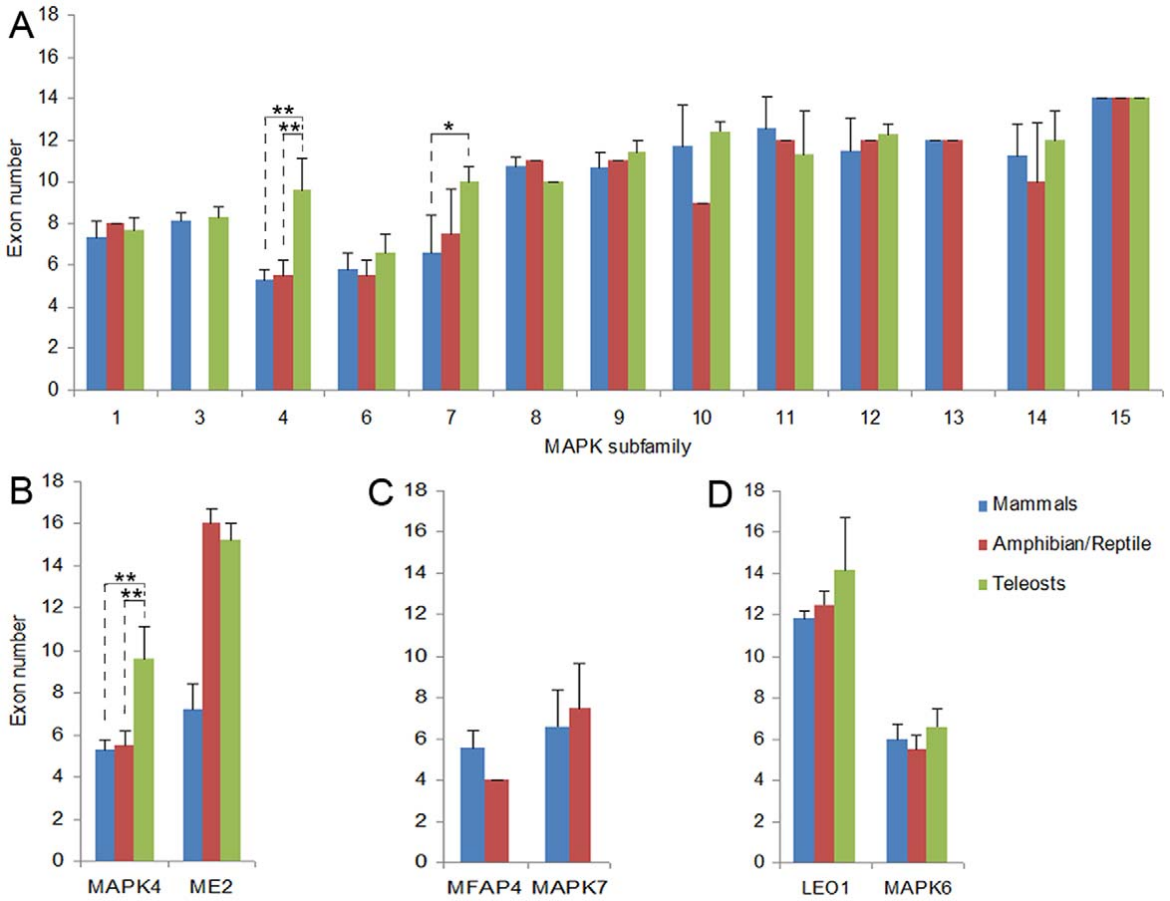
Exon numbers of the vertebrate MAPK subfamilies. The average exon numbers of 13 vertebrate MAPK subfamilies are shown in panel (**A**). The comparisons of MAPK4, MAPK7, and MAPK6 with their flanking genes in average exon numbers are shown in panels (**B**), (**C**), and (**D**), respectively. In panel (**B**), only MAPK4 and its right-flanking (ME2) genes were taken into account due to a difference of its left-flanking genes between teleosts and amphibians (Figure S4), and in panel (**D**), only MAPK6 and its left-flanking (LEO1) genes were taken into account due to a difference of its right-flanking genes between teleosts and amphibians (Figure S4). In panel (**C**), only MAPK7 and its left-flanking (MFAP4) genes from amphibians and mammals were taken into account due to differences of both its left- and right-flanking genes between teleosts and other taxa, and of its right-flanking genes between amphibians and mammals (Figure 3). One asterisk indicates P<0.05 and two asterisks P<0.01 (one-way ANOVA followed by the Bonferroni test).

To further understand the evolutionary history of MAPK family, we analyzed the exon-intron structure of each vertebrate MAPK gene. The exon numbers of the vertebrate MAPK subfamilies ranged from 5 to 14 (Figures 4A and S5). The exon/intron numbers of most members were similar to each other in certain MAPK subfamily, consistent with their phylogenetic classification. In both MAPK1 and MAPK3 subfamilies, the numbers were mainly 8 with a range from 6 to 9. In the JNK (MAPKs 8–10) subfamilies, the exon numbers had a range from 9 to 14. Except the OanMAPK12 (7 exons) and the CluMAPK11 (16 exons), the P38 (MAPKs 11–14) subfamilies had 9 to 13 exons (Figures 4A and S5). The similarities in the exon numbers between MAPK subfamilies 8–14 supported that they had arisen from one common ancestor by gene duplication (Figures 2 and 4A) [33].

The MAPK subfamilies 4, 6 and 15 had closer evolutionary relationship to each other than to the other subfamilies and shared one earlier common progenitor (Figures 1 and 2). Intriguingly, among the MAPK family, the MAPK15 subfamily had the maximum exon/intron numbers, whereas the MAPKs 4&6 subfamilies exhibited the minimum exon/intron numbers. The exons (approximately 14) possessed by most MAPK15 genes were significantly more than that possessed by most MAPK4 (about 7 exons, P<0.01) and MAPK6 (about 6 exons, P<0.001) genes. Furthermore, the CinMAPK4 at root of the MAPKs 4&6 subclade had 13 exons (Figure S5). These suggest that intron loss had occurred in both MAPK4 and MAPK6 genes during the long-term evolution. In particular, in the MAPK4 subfamily, the teleosts had obviously more exons than other species (P<0.01) (Figure 4, A and B). Therefore, we further investigated whether the exon numbers of other MAPK subfamilies differed between species by comparing the exon numbers between teleosts, amphibians/reptiles and mammals. We found that the MAPK7 subfamily also exhibited obviously different exon numbers between the three vertebrate taxa, whereas all other MAPK subfamilies (1, 3, 6 and 8–15) had conserved exon numbers between three vertebrate taxa (Figure 4A). For the MAPK4 subfamily, both mammals (about 5 exons) (P<0.01) and amphibians (about 6 exons) (P<0.01) possessed significantly fewer exons than teleosts (about 10 exons) (Figure 4A), implying that intron loss events continually occurred in MAPK4 during the evolutionary transition from teleosts to amphibians and mammals. For the MAPK7 subfamily, the mammals (about 6 exons) appeared to have significantly fewer exons than amphibians (about 10 exons) (P<0.05) and teleosts (about 10 exons) (P<0.05) (Figure 4A), indicating that MAPK7 experienced intron loss during the evolutionary transition from teleosts to amphibians and mammals.

To determine whether these intron losses were MAPK gene-specific, we also compared the exon numbers of the flanking genes of MAPK4 and MAPK7 genes between three vertebrate taxa, teleosts, amphibians and mammals. For the MAPK4 genes, because the left-flanking genes were different between teleosts and amphibians (Figure S4), only the right-flanking genes (ME2) were taken into account. Compared with the MAPK4 genes that had a significant decrease in exon numbers during the transition from teleosts to amphibians, the ME2 genes did not show the decrease (Figure 4B). For the MAPK7 genes, because both the left- and right-flanking genes were different between teleosts and other taxa, and the right-flanking genes were different between amphibians and mammals (Figure 3C), only the left-flanking genes (MFAP4) from amphibians and mammals were taken into account. Distinct from the MAPK7 genes, MFAP4 genes did not show an obvious decrease in exon numbers from amphibians to mammals (Figure 4C). These results indicate that the intron loss events occurring in the MAPK4 and MAPK7 genes did not simultaneously happen not only in their flanking genes (Figure 4, B and C), but also in other MAPK genes (Figure 4, A and D, and data not shown). Intron loss had been demonstrated to enhance the level of gene expression [34]. Our results presumed that the expressions of the MAPK4 and MAPK7 genes might be enhanced in mammals via intron loss, compared with their counterparts in teleosts and amphibians, respectively.

### Purifying selection acting on the vertebrate MAPK genes

**Figure 5:**
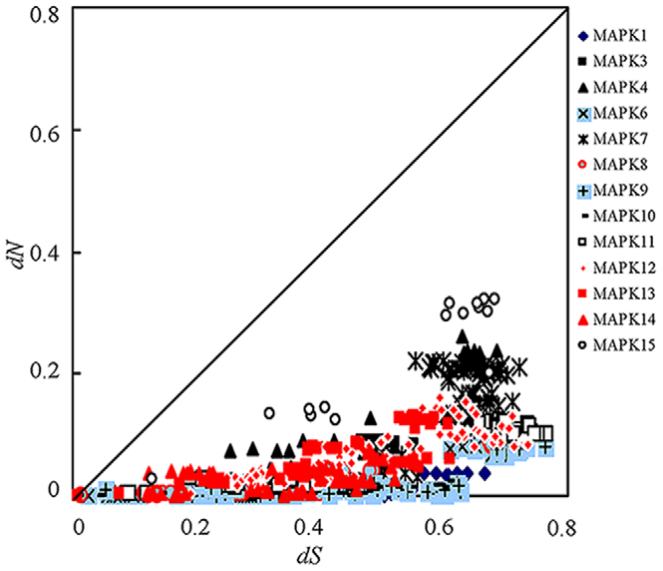
Pairwise comparison plots of *dN* and *dS* values for each MAPK subfamily. The *dN* and *dS* values were calculated with MEGA4.0. The transition/transversion ratios used for each subfamily are: MAPK1: 2.398; MAPK3: 4.008; MAPK4: 1.428; MAPK6: 2.183; MAPK7: 1.167; MAPK8: 1.132; MAPK9: 1.745; MAPK10: 2.126; MAPK11: 1.706; MAPK12: 1.552; MAPK13: 0.809; MAPK14: 1.803; MAPK15: 1.361.

The non-synonymous to synonymous rate ratio ω (*dN*/*dS*) is an indication of the change of selective pressures. The ω ratios of <1,  = 1 and >1 indicate purifying selection, neutral evolution and positive selection on the gene involved, respectively. Pairwise comparisons of *dN* and *dS* within each MAPK subfamily using MEGA 4 showed that almost all *dN*/*dS* rate ratios were significantly lower than 1 (p<0.01, Z-test, Figure 5), indicating purifying (negative) selection acting on MAPK family. However, the protein alignment reveals high sequence variation among the vertebrate MAPK subfamilies (Figures S2 and S3). To examine whether individual amino acid sites of MAPK proteins are under positive selection, we tested for variable ω rate ratios among various lineages using the free-ratio model implemented in PAML 4.0 [35]. In the analyses of MAPK subfamilies 1, 6, 8, 9, 11, 12 and 13, the codon substitution free-ratio model (model  = 1, M1) that allows different ω ratios among branches did not fit the data better than the model that assumes a homogeneous mean ω rate ratio for all lineages (model  = 0, M0) (Table S2). The estimates of ω for these MAPK subfamilies (0.002–0.052) were substantially smaller than 1. For other MAPK subfamilies (3, 4, 7, 10, 14, and 15), the free-ratio model fitted the data better than the one-ratio model, suggesting that different lineages of these MAPK subfamilies experienced variable selective pressures. Therefore, we further used the site-model to examine whether positive selection drove the evolution of the MAPK family. No significant evidence of positive selection was detected in each MAPK subfamily (ω<<1) (Table 2), supporting that purifying selection drove the evolution of the MAPK family.

**Table 2:** Site-model (M7 vs. M8) test for each MAPK gene subfamily.

Subfamily	dN/dS (M7)	Estimates of parameters		lnL		2Δl	P-value
		M7	M8	M7	M8		
MAPK1	0.0024	p = 0.07 q = 19.51	p0 = 1.00(p1 = 0.00) p = 0.13 q = 69.24 ω = 1.00	−3134.47	−3133.72	1.4972	0.4730
MAPK3	0.0394	p = 0.11 q = 2.26	p0 = 1.00(p1 = 0.00) p = 0.11 q = 2.26 ω = 1.00	−4441.69	−4441.69	0.0031	0.9985
MAPK4	0.0641	p = 0.34 q = 4.72	p0 = 1.00(p1 = 0.00) p = 0.34 q = 4.72 ω = 5.38	−5993.08	−5993.09	0.0064	0.9968
MAPK6	0.0207	p = 0.17 q = 6.93	p0 = 1.00(p1 = 0.00) p = 0.17 q = 6.93 ω = 1.00	−4770.52	−4770.52	0.0063	0.9969
MAPK7 a	0.0368	p = 0.33 q = 6.91	p0 = 0.99(p1 = 0.01) p = 0.36 q = 8.43 ω = 1.00	−11585.8	−11582.68	6.2322	**0.0443**
MAPK8	0.0253	p = 0.15 q = 5.13	p0 = 1.00(p1 = 0.00) p = 0.15 q = 5.13 ω = 1.00	−3284.55	−3284.55	0.0023	0.9989
MAPK9	0.0120	p = 0.16 q = 11.23	p0 = 1.00(p1 = 0.00) p = 0.16 q = 11.23 ω = 2.79	−3579.26	−3579.27	0.0042	0.9979
MAPK10	0.0126	p = 0.03 q = 1.45	p0 = 1.00(p1 = 0.00) p = 0.03 q = 1.45 ω = 1.00	−4026.99	−4026.99	0.0056	0.9972
MAPK11	0.0225	p = 0.19 q = 7.19	p0 = 1.00(p1 = 0.00) p = 0.19 q = 7.19 ω = 3.58	−3333.29	−3333.3	0.0045	0.9978
MAPK12	0.0343	p = 0.23 q = 5.87	p0 = 1.00(p1 = 0.00) p = 0.23 q = 5.87 ω = 1.00	−2825.32	−2825.32	0.0017	0.9991
MAPK13	0.0663	p = 0.28 q = 3.65	p0 = 0.99(p1 = 0.01) p = 0.31 q = 4.77 ω = 1.04	−4741.85	−4739.72	4.2685	0.1183
MAPK14	0.0467	p = 0.20 q = 3.73	p0 = 1.00(p1 = 0.00) p = 0.20 q = 3.73 ω = 4.34	−4402.47	−4402.47	0.0056	0.9972
MAPK15	0.1647	p = 0.43 q = 2.12	p0 = 1.00(p1 = 0.00) p = 0.43 q = 2.12 ω = 1.00	−6287.07	−6287.07	0.0001	1.0000

lnL: the log-likelihood difference between the two models; 2Δl: twice the log-likelihood difference between the two models.

a In the analysis of MAPK subfamily 7, the P-value is less than the significance level 0.05, indicating that the M8 model fitted the data better than M7 model. However, the estimate of ω in M8 was less than (99% sites) or equal to 1 (1% sites), indicating no positive selection.

### Highly conserved motifs in the vertebrate MAPK subfamilies

**Figure 6:**
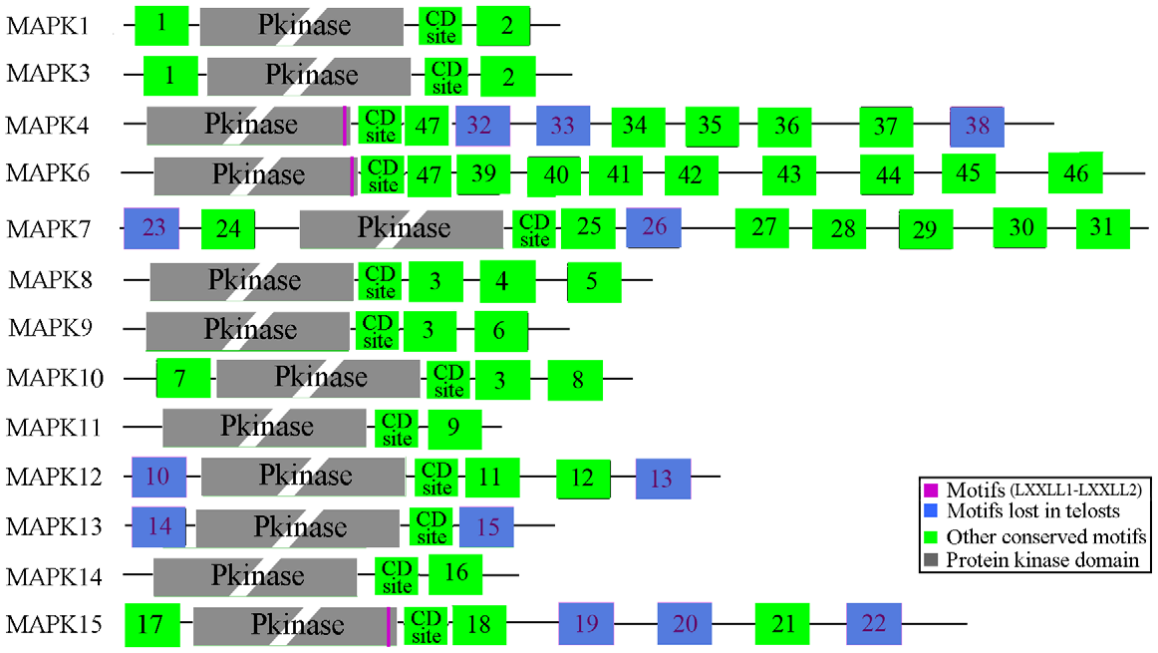
Motif distributions of the 13 vertebrate MAPK subfamiles. The protein kinase domains are drawn as grey boxes. The motifs lost in teleosts are drawn as blue and other motifs as green. The LXXLL1-LXXLL2 motifs are marked in purple. The motifs, especially the protein kinase domains, are not drawn to scale. The sequences of these motifs are given in Table S3.

We queried the PFAM database of protein domains with the MAPK proteins and identified a significant match to the protein kinase domain in all the MAPK family members (Figure 6), consistent with previous identification of many conserved amino acids locating around the MAPKs catalytic cleft [36], [37]. We then investigated the amino acid patterns and newly characterized 47 motifs out of the protein kinase domain and the conserved common docking site (CD site) in the 13 MAPK subfamily members (Figure 6 and Table S3) [38], [39], [40], [41]. None of the 47 motifs could be found in the PFAM database.

**Figure 7:**
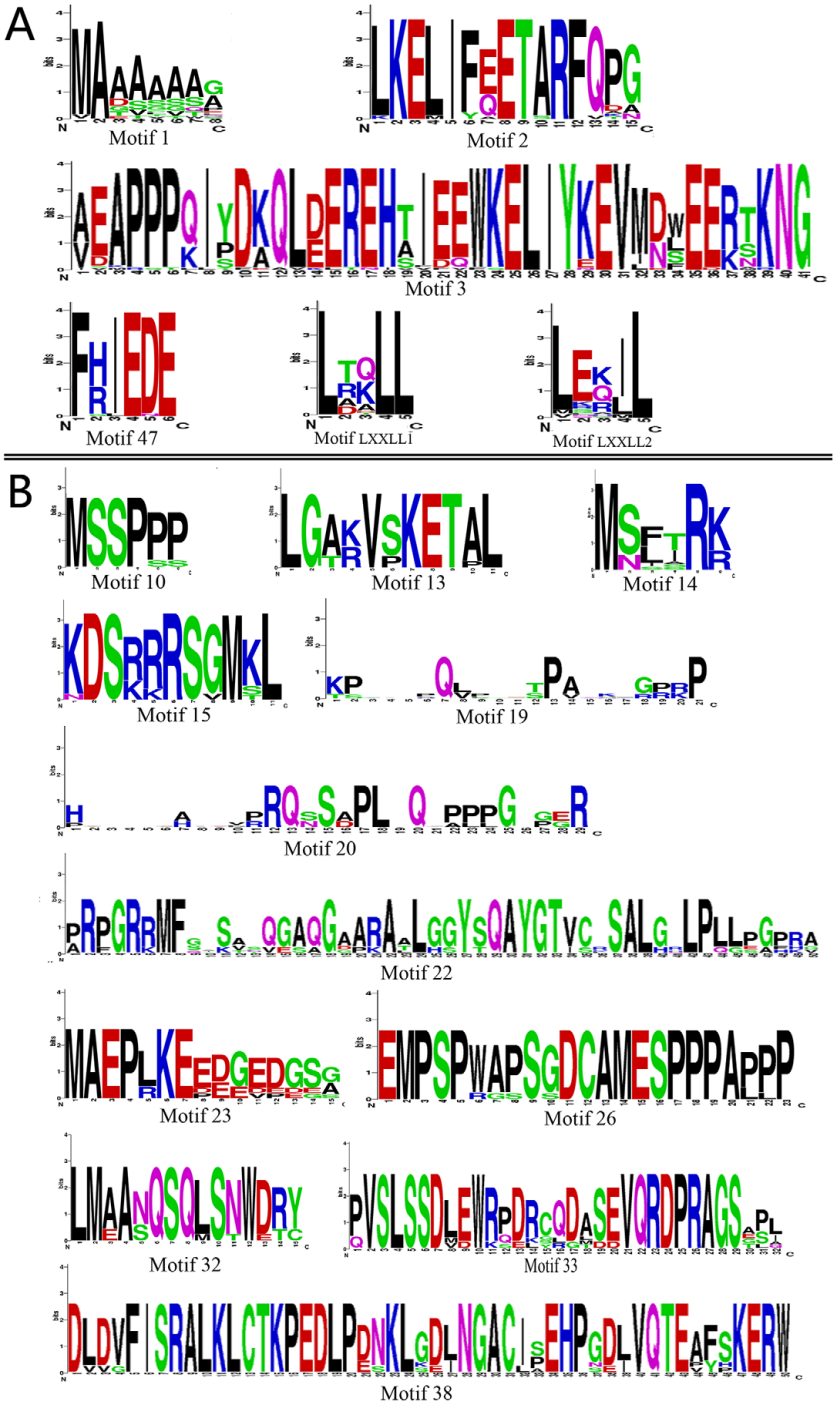
Sequence logos of some specific motifs identified in this study. (**A**), the motifs shared by at least two vertebrate MAPK subfamilies. (**B**), the motifs specially found in higher vertebrate taxa. The character and size of each logo represent the proportion of an amino acid at the specific site. The sequence logos were generated by the web-based program WEBLOGO 3. The sequence logos of other motifs are shown in Figure S6.

Among these motifs, most (40/47, 85%) were located downstream of the protein kinase domain (i.e. the C-terminus of the MAPK protein). Only one motif was identified at the N-terminus (i.e. the upstream of the protein kinase domain) of MAPK proteins in subfamilies 1, 3, 10, 12, 13, and 15, two motifs in subfamily 7, and no motif in subfamilies 4, 6, 8, 9, 11, and 14 (Figure 6) . In addition, we noted that most MAPK subfamilies contained less than 7 motifs, whereas the MAPK subfamilies 4 (8 motifs), 6 (9 motifs), and 7 (9 motifs) possessed more than 7 motifs (Figure 6). The C-terminus has been demonstrated to be critical to the functions of the MAPK6 and MAPK7 [24], [37], [42], [43], [44], [45], [46]. Therefore, the C-terminal tail of the MAPK4 and even other MAPK subfamily members were suggested to also be important for their function. On the other hand, almost all motifs (except motifs 1, 2, 3, and 47) only appeared in one MAPK subfamily, suggesting that they might be associated with the MAPK subfamily-specific functional divergence (Figures 6 and 7). Motifs 1 and 2 were shared by the MAPK subfamilies 1 and 3, motif 3 by MAPK subfamilies 8, 9 and 10, and motif 47 by MAPK subfamilies 4 and 6 (Figures 6 and 7). In addition, two LXXLL motifs (typical of agonist-bound nuclear receptor corepressors) that were specifically required for the MAPK15 to interact with ERRα and to regulate its cellular localization and transcriptional activity were demonstrated to be perfectly conserved down to *Drosophila melanogaster* [47]. Although not being detected by the MEME/MAST test here, the two LXXLL motifs were found to be conserved in MAPK subfamilies 4, 6 and 15 by sequence comparison (Figures 6 and 7, Table S3). Interestingly, some motifs appearing in higher vertebrate taxa did not exist in teleosts (Figure 6), suggesting that they might contribute to the functional divergence of MAPK subfamilies between teleosts and mammals or other higher vertebrate taxa. A case in point was that both the MAPK4 and MAPK15 in mammals possessed 3 additional motifs, compared with their homologs in teleosts (Figure 6). The additional 3 motifs were located on the C-terminal tail of MAPK4 and MAPK15. The C-terminal tail is important for MAPK4 and MAPK15 functions. For example, the C-terminal tail of MAPK15 regulates not only its kinase activity by a way independent from the extracellular signal-mediated activation stimuli [42], but also its nuclear localization and the inhibition of cell growth [24]. Therefore, these motifs specially found in higher vertebrate taxa might be useful for investigating the functions of MAPK4 and MAPK15.

## Discussion

The MAPK signaling cascade is involved in various cellular processes and is well conserved in evolution [10], [11], [16], [24]. In the vertebrate MAPK family, 13 members have been previously identified. However, the evolutionary history of these members remains largely unclear. In this study, we collected the MAPK genes from vertebrates, invertebrates and plants to draw the most comprehensive evolutionary scenario of the vertebrate MAPK family. We found that vertebrates had substantially more MAPK family members than invertebrates (Table 1), and the vertebrate MAPK family had been formed through multiple duplications at least prior to the diversification of teleosts (Figure 1). Intriguingly, after rapid extensive gene duplication events, not all vertebrates have all 13 MAPK family members (Figure 1). This might be attributed to two reasons. First, the genome sequences of the vertebrates analyzed were incomplete. Second, gene loss events might have happened in some vertebrate species, as observed in amphibians and teleosts, both of which lost some MAPK family members (Figure 1). These results suggest that a burst of gene duplication events and subsequent widespread gene deletion events [48], had occurred during the early evolution of the vertebrate MAPK family.

In the phylogenetic analyses, six subclades were found, including MAPKs 1&3, MAPKs 4&6, MAPK7, MAPK15, JNK (MAPKs 8–10) and P38 (MAPKs 11–14) (Figure 2). The six subclades were consistent with previous classification of the MAPK family [2], [11], [26]. To thoroughly investigate the evolutionary origin of the MAPK family, we added more MAPK genes from some invertebrates and plants into our phylogenetic analyses. The results show that the vertebrate MAPK subfamilies were duplicated from 3 earlier progenitors (Figure 2). It has been suggested that the multiplicity of the mammalian kinases has arisen partly by two rounds of genome duplications [33], [49]. However, our results show that except for the subclade of P38 (MAPKs 11–14) and JNK (MAPKs 8–10), the pattern of two rounds of genome duplications seemed not to be supported by the evolutionary relationships of the other vertebrate MAPK subfamily members. The MAPKs 4&6 subgroup had been previously thought to be exclusive for chordates and vertebrates [26]. However, the identification of the orthologs of MAPK4 or MAPK6 in *Ciona intestinalis* (Figure 2) suggests that the origin of the MAPKs 4&6 subfamilies should have predated the emergence of the common ancestor of echinoderms and chordates, more than 550 million years ago [31].

Comparative analyses of the MAPK family synteny show that a conserved block of genes next to each MAPK family member had been maintained throughout the vertebrate diversification (Figures 3 and S4). The P38 (MAPKs 11–14) subfamilies were the only subclade of MAPK family located tandemly on two different chromosomes (i.e. MAPK11 and MAPK12 in one chromosome, and MAPK13 and MAPK14 in another one). The close phylogenetic relationships between MAPK11 and MAPK14, and between MAPK12 and MAPK13 (Figures 1 and 2) indicate that both MAPK11 and MAPK14 were a pair of duplicated sister genes, and both MAPK12 and MAPK13 were another pair, thereby suggesting that a segmental duplication event led to both MAPK11 and MAPK12 in one chromosome and both MAPK13 and MAPK14 in another one. In addition, the MAPK subfamilies 11 and 14 had close genetic relationship with the ancestral MAPK genes of invertebrates, and they existed more extensively among teleosts, amphibians and mammals than the MAPK subfamilies 12 and 13 (Figure 2). These suggest that MAPK12 arose from a tandem duplication of MAPK11 and formed a gene unit with MAPK11, and the MAPK13-MAPK14 gene unit originated from a segmental duplication of the gene unit of MAPK11-MAPK12.

Purifying selection was detected in all MAPK subfamilies (Table 2, Figure 5), indicating strong functional constraints of MAPK genes. Intriguingly, however, we found that the MAPK subfamilies 4 and 7 had experienced intron loss during the evolutionary transitions from teleosts to amphibians and from amphibians to mammals, respectively, whereas their flanking genes did not (Figure 4). Intron loss had been demonstrated to be able to enhance the level of gene expression [34]. The intron losses specifically occurring on the MAPK4 and MAPK7 genes might be the result of the adaptive evolution of vertebrates, which might be associated with a transition from teleosts to amphibians and mammals.

On the other hand, we found that most motifs only appeared in one MAPK subfamily (Figure 6), implying an association with subfamily-specific functional divergence of MAPK. In particular, we found that MAPK subfamilies 4, 6 and 7 possessed more motifs than other MAPK family members, and most of these motifs were located at the C-terminus, downstream of the protein kinase domain (Figure 6). This indicates that the C-terminus might be critical to the functions of MAPK 4, 6, and 7 [24], [37], [42], [43], [44], [45], [46]. In addition, three motifs 32, 33, and 38 in the MAPK4 subfamilies, and three motifs 19, 20, and 22 in the MAPK15 subfamilies were found to be individually gained in higher vertebrates after the divergence from teleosts (Figure 6). These newly gained motifs might play potential roles in adaptive evolution of these higher vertebrates.

## Materials and Methods

### Sequence data

To gain a full list of MAPKs in vertebrate, the BLASTP and TBLASTN programs with the known MAPK sequences as queries were used to search mammalian and avian genome assemblies from GenBank, including *Homo sapiens, Macaca mulatta, Pan troglodytes, Mus musculus, Rattus norvegicus, Ornithorhynchus anatinus, Monodelphis domestica, Bos taurus, Canis lupus familiaris, Equus caballus, Ovis aries, Sus scrofa, Gallus gallus* and *Taeniopygia guttata*. With the HMM profile built based on those reported vertebrate MAPKs, HMMsearch [50] was used to screen the Ensembl genome assemblies (http://www.ensembl.org/index.html) of *Gasterosteus aculeatus, Oryzias latipes, Takifugu rubripes, Tetraodon nigroviridis, Anolis carolinensis, Xenopus tropicalis and Tursiops truncatus*. HMMsearch were also performed on the genome assemblies of *Ciona intestinalis* [31], *Ciona savignyi* and *Strongylocentrotus purpuratus* [51]. Both *Arabidopsis thaliana* and *Oryza sativa* MAPK sequences were retrieved from GenBank. The zebrafish (*Danio rerio*) MAPK sequences reported by Krens et al. [29] were downloaded from GenBank. MAPK sequences of nematodes (*Caenorhabditis briggsae, Caenorhabditis remanei* and *Caenorhabditis elegans*) and arthropods (*Apis mellifera, Drosophila melanogaster, Ixodes scapularis, Aedes aegypti and Acyrthosiphon pisum*) were respectively retrieved from wormbase (http://www.wormbase.org/) and GenBank or Flybase (http://flybase.org/) by the BLAST program. All of those searches were performed from March to July 2010.

### Multiple sequence alignment and phylogenetic tree reconstruction

All the MAPK protein sequences were aligned using the L-INS-I method (iterative refinement method that incorporates local and pairwise alignment information) implemented in MAFFT v6.6 [52], with the following parameters: scoring matrix for amino acid sequences, BLOSUM62; gap opening penalty, 2.0; and gap extension penalty, 0.2. The protein kinase domain alignment was then manually refined and end trimmed to eliminate the poorly aligned positions and divergent regions (*e.g.* a number of indels and mismatches) using BioEdit version 7.0.5.3 (http://www.mbio.ncsu.edu/BioEdit/BioEdit.html). Unambiguously aligned positions were used for the subsequent phylogenetic analyses. A maximum likelihood (ML) phylogeny was reconstructed for the MAPK protein family with PHYML v2.4 [53]. The JTT+I+G model was selected as the best-fitting amino acid substitution model for ML analysis according to the Akaike information criterion in ProtTest [54]. The Bayesian analysis was performed with MrBayes version 3.1.2 [55], [56]. Two independent runs were computed for 6 million generations, at which point the standard deviation of split frequencies was less than 0.01, and one tree was saved every 100 generations, and 45,000 trees from each run were summarized to give rise to the final cladogram.

### Molecular evolutionary analyses

To detect whether positive selection acts on MAPK family, the numbers of non-synonymous substitutions per nonsynonymous site (*dN*) and that of synonymous nucleotide substitutions per synonymous site (*dS*) were computed using the modified Nei–Gojobori method in MEGA 4.0. The significance of the difference between *dN* and *dS* was estimated with the codon-based Z-Test in MEGA 4.0. Because Codon-based substitution models are routinely used to identify amino acid sites under positive selection, the program CODEML implemented in PAML 4 [35] was used to test the MAPK family for positive selection. The codon alignments of MAPK genes were generated by PRANK that is demonstrated to be more accurate than other alignment methods in the test of positive selection [57]. A significant difference in the ω rate ratio (the nonsynonymous to synonymous substitution rate ratio, also known as *dN/dS*) between different branches was tested by comparing a free-ratio model (model  = 1), which allows ω to vary along different branches, with a model assigning a mean ratio to all lineages (model  = 0). For simplicity, site-specific model was performed on MAPK families by comparing the selection model M8 with the null model M7. M7 assumes a beta distribution of ω values between 0 and 1, and does not allow for sites under positive selection. M8 is similar to M7 but has another category of sites in which ω>1. Likelihood ratio tests (LRT) of different models were used to find the best fit model for the data. Statistical significance was evaluated by comparing twice the log likelihood difference between models to a chi-square statistic with the degrees of freedom equal to the difference in number of parameters between models.

### Conserved domain and motif detection

The domain architecture of MAPK amino acid sequences was predicted by searching against the PFAM 23.0 database (http://pfam.sanger.ac.uk/) with the HMMPFAM program implemented in HMMER2.3.2 [50]. The MEME and MAST software (http://meme.sdsc.edu/meme/website/intro.html) [58] were used to investigate conserved motifs in the complete amino acid sequences of MAPK proteins.

## Supporting Information

Figure S1 **The phylogeny of the species involved in this study.** This tree was reconstructed referring to the species tree in Ensembl (http://asia.ensembl.org/info/about/species.html).
(TIF)

Figure S2 **Complete amino acid sequence alignment of the vertebrate MAPK family.** This alignment was used to reconstruct the ML phylogenetic tree shown in Figure 1.
(PDF)

Figure S3 **Complete amino acid sequence alignment of MAPKs from vertebrates, tunicates, echinoderm, nematodes, arthropods and plants.** This alignment was used to reconstruct the Bayesian phylogenetic tree shown in Figure 2.
(PDF)

Figure S4 **Order and orientation of genes syntenic to MAPK4 (A) and MAPK6 (B).**
(PDF)

Figure S5 **Gene structure of the 13 vertebrate MAPK subfamilies.**
(DOC)

Figure S6 **Sequence logos of other motifs identified in this study.** For details, see Table S3.
(PDF)

Table S1 **List of the vertebrate MAPK homologs identified in the genomes of selected species.**
(XLS)

Table S2 **Free model test for each MAPK subfamily.**
(DOC)

Table S3 **Sequences of the motifs identified in the MAPK family.**
(XLS)
