# Supplementary figures and images for: Evolutionary History of the Vertebrate Mitogen Activated Protein Kinases Family
Source: PLoS One. 2011 Oct 26;6(10):e26999. doi: 10.1371/journal.pone.0026999 (PMC3202601; doi:10.1371/journal.pone.0026999)

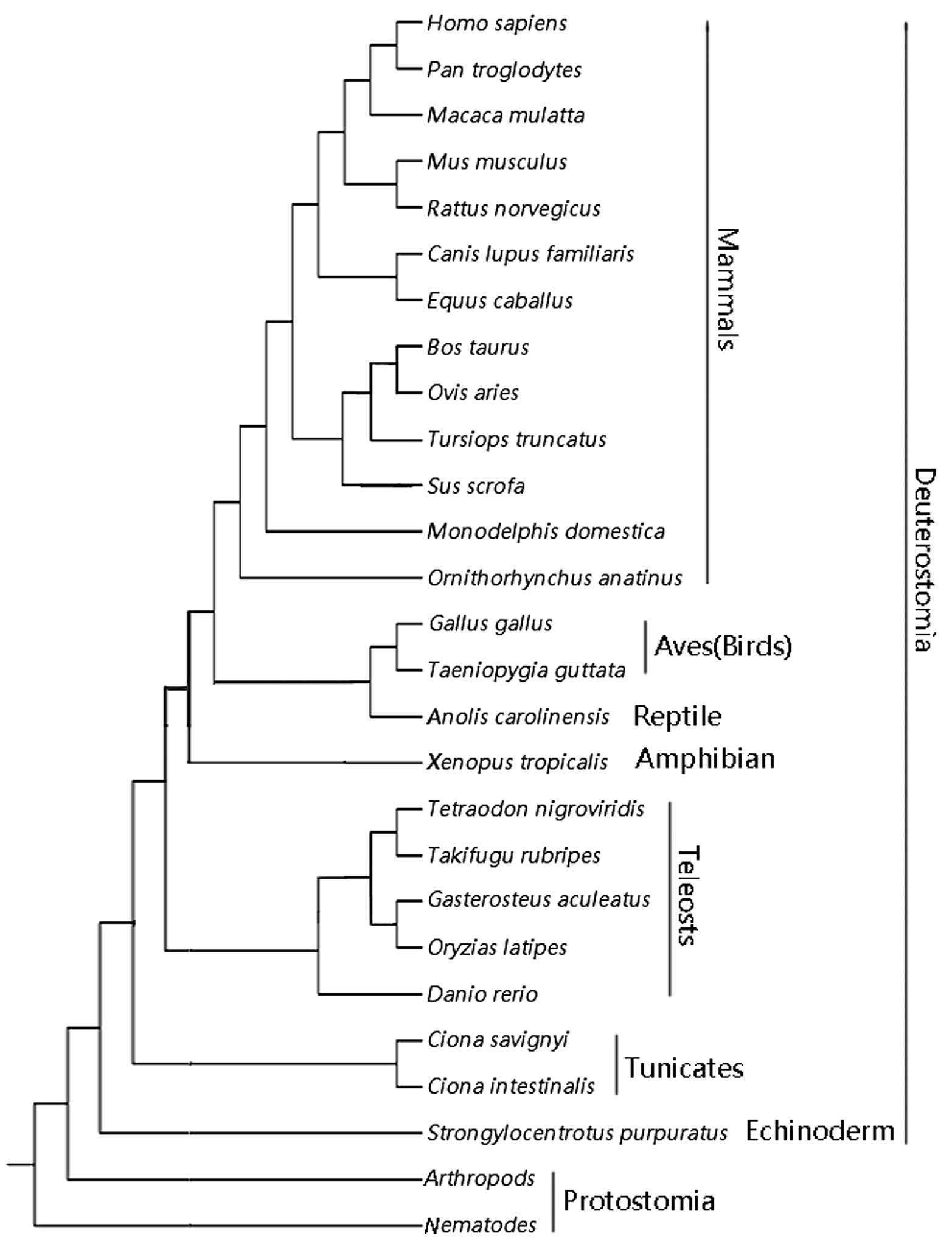

Supplement: Figure S1 — The phylogeny of the species involved in this study. This tree was reconstructed referring to the species tree in Ensembl (http://asia.ensembl.org/info/about/species.html). (TIF) [file pone.0026999.s001.tif]

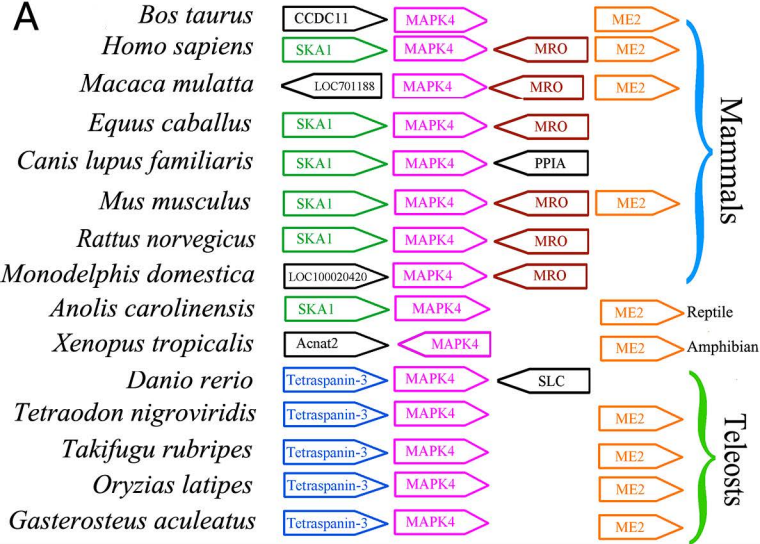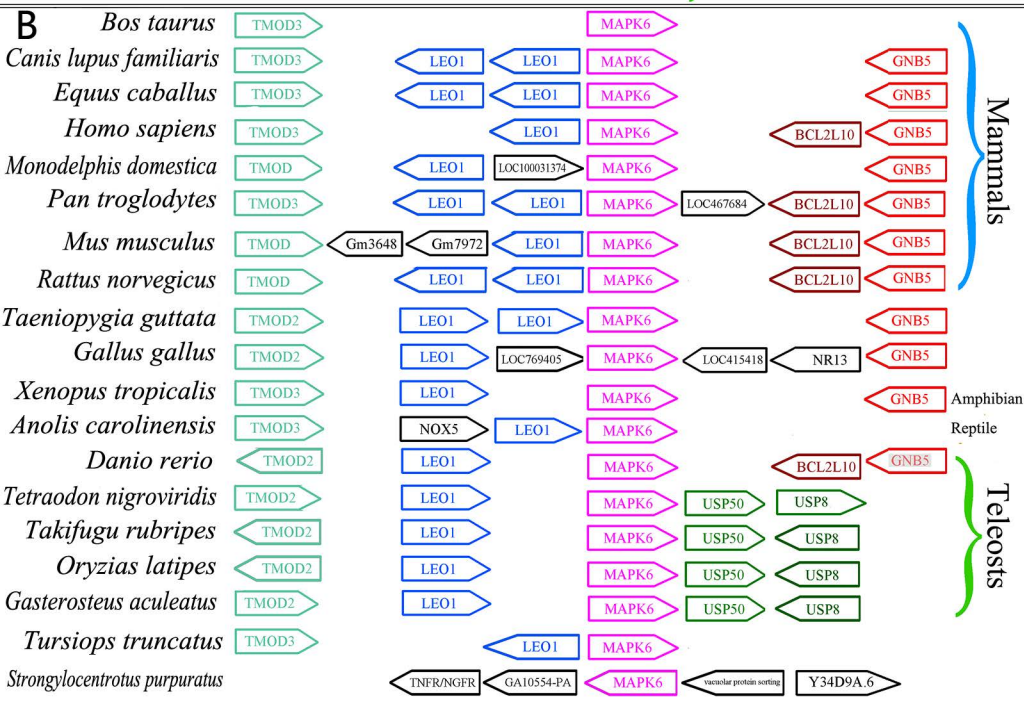

Supplement: Figure S4 — Order and orientation of genes syntenic to MAPK4 (A) and MAPK6 (B). (PDF) [file pone.0026999.s004.pdf]

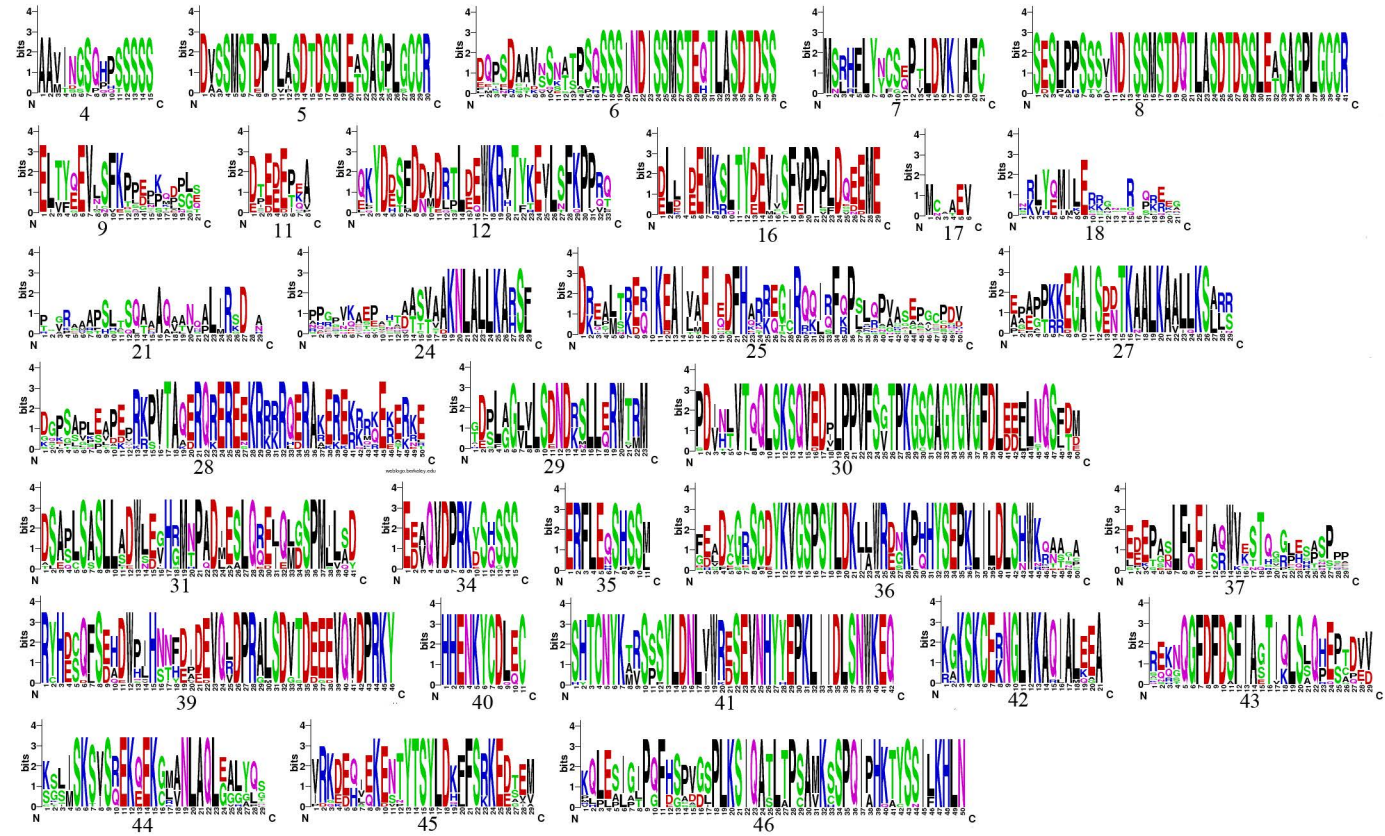

Supplement: Figure S6 — Sequence logos of other motifs identified in this study. For details, see Table S3. (PDF) [file pone.0026999.s006.pdf]
