# Supplementary material for: Evolutionary History of the Vertebrate Mitogen Activated Protein Kinases Family
Source: PLoS One. 2011 Oct 26;6(10):e26999. doi: 10.1371/journal.pone.0026999 (PMC3202601; doi:10.1371/journal.pone.0026999)
Supplement: Figure S5 — Gene structure of the 13 vertebrate MAPK subfamilies. (DOC) [file pone.0026999.s005.doc]

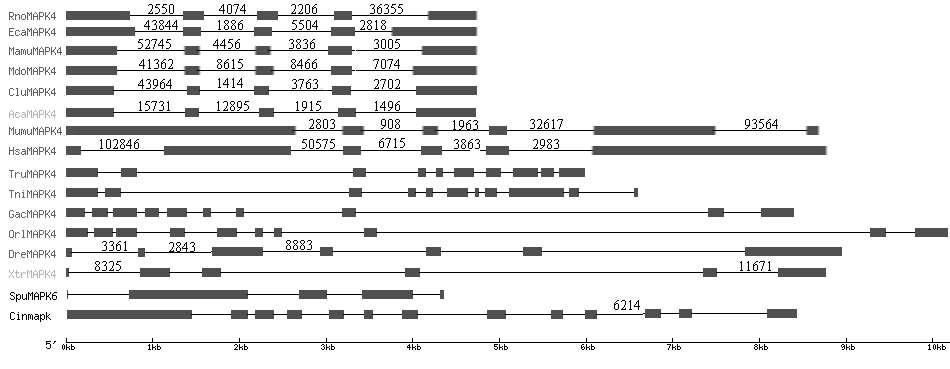


**MAPK4 subfamily.**


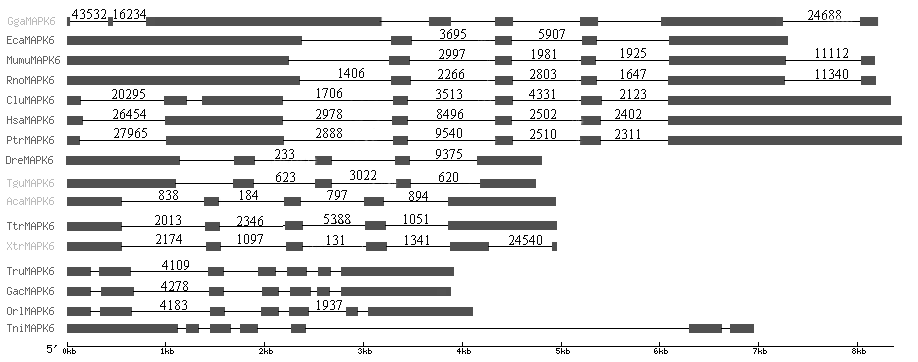


**MAPK6 subfamily**


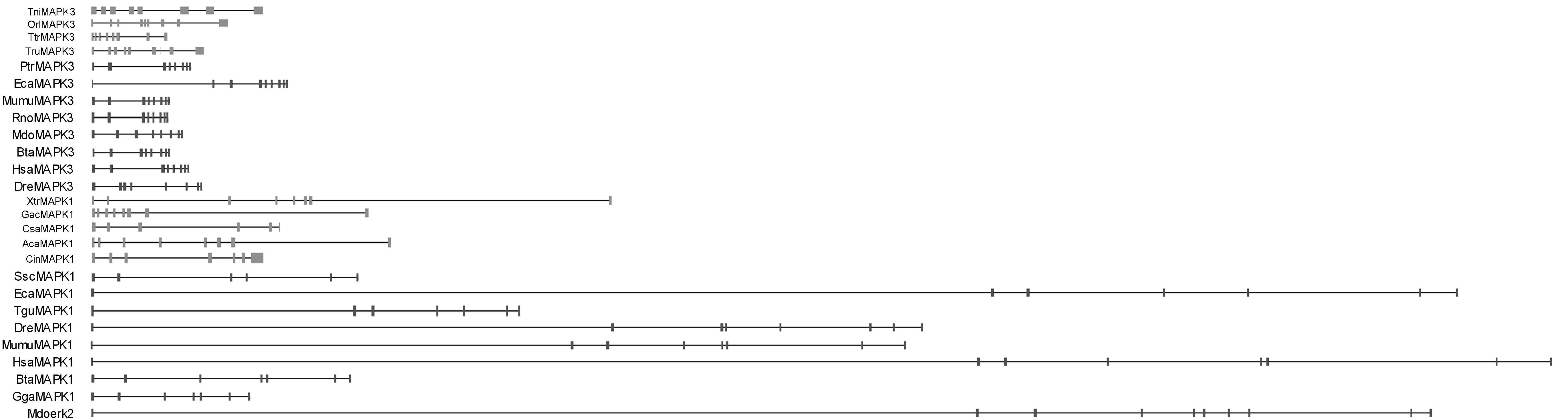


**MAPK1 and MAPK3 subfamilies**


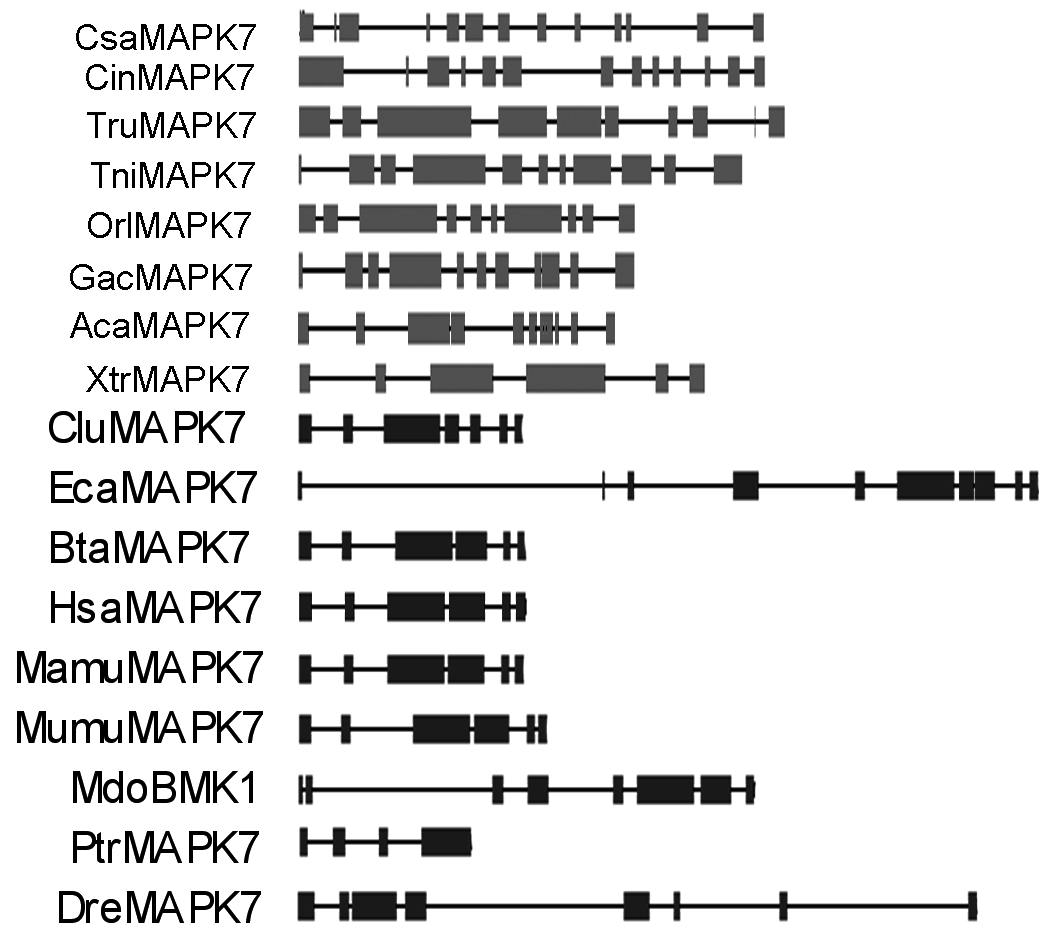


**MAPK7 subfamily**


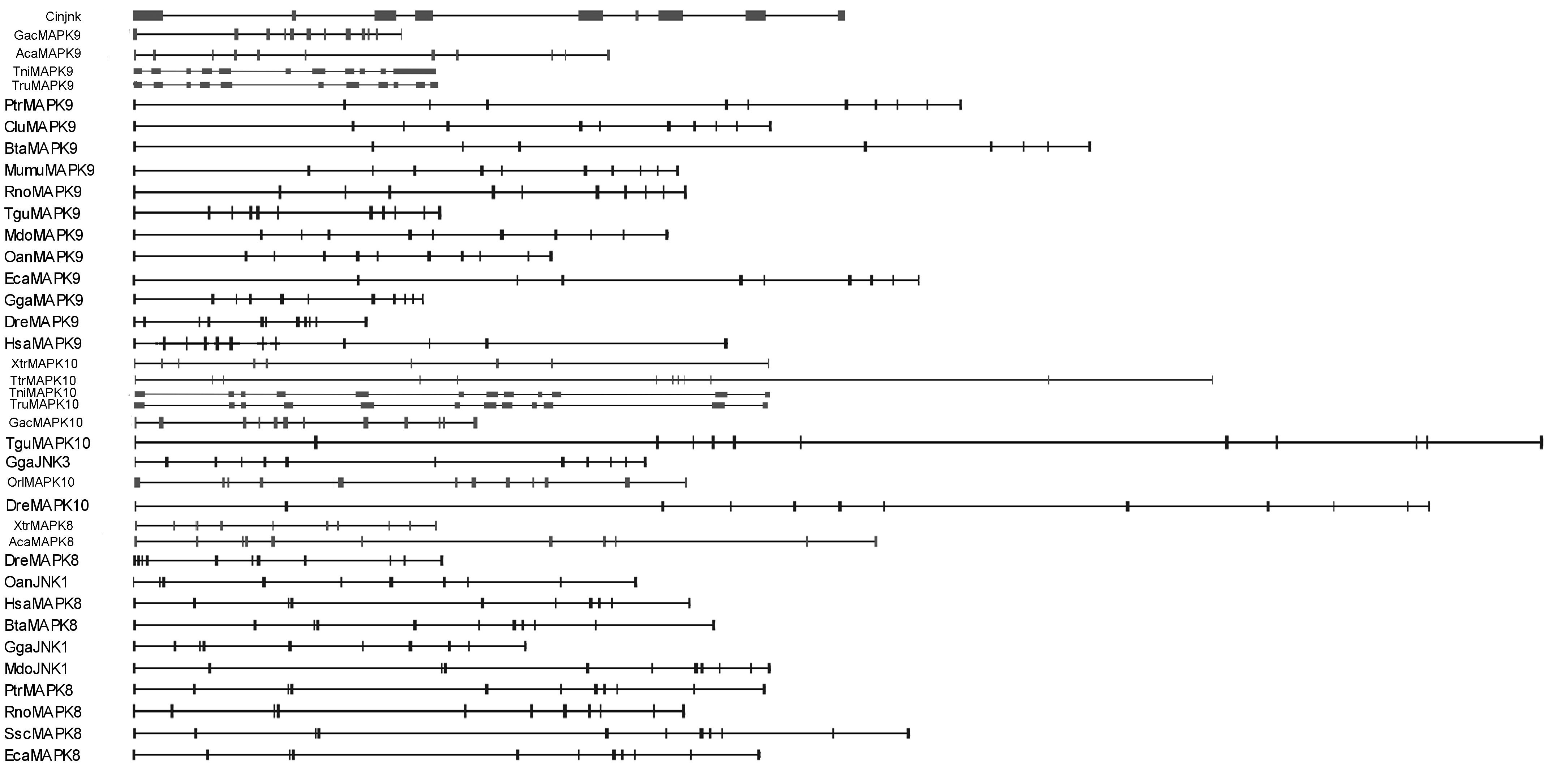


**JNK (MAPK8-10) subfamilies**


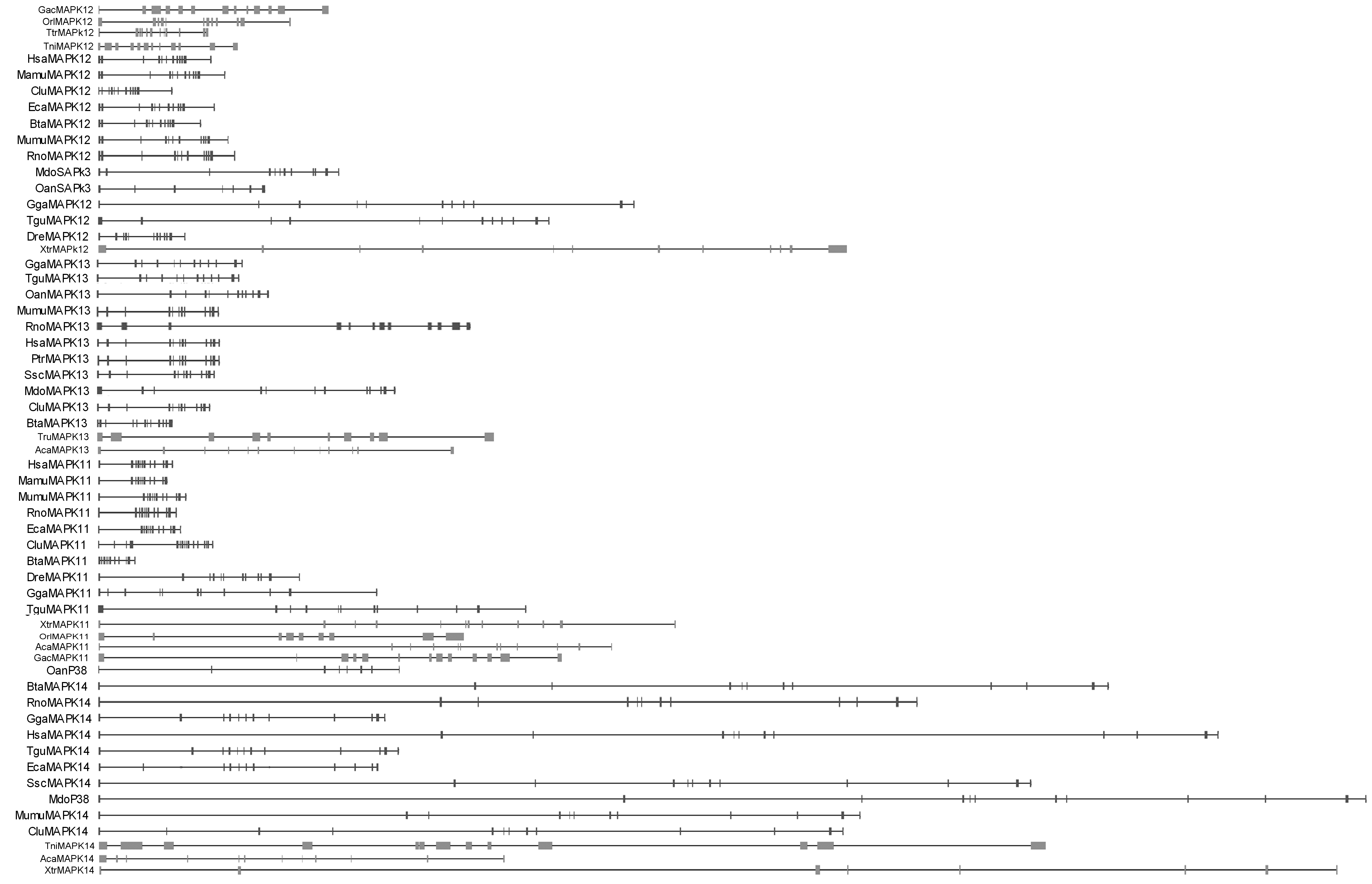


**P38 (MAPK11-14) subfamilies**


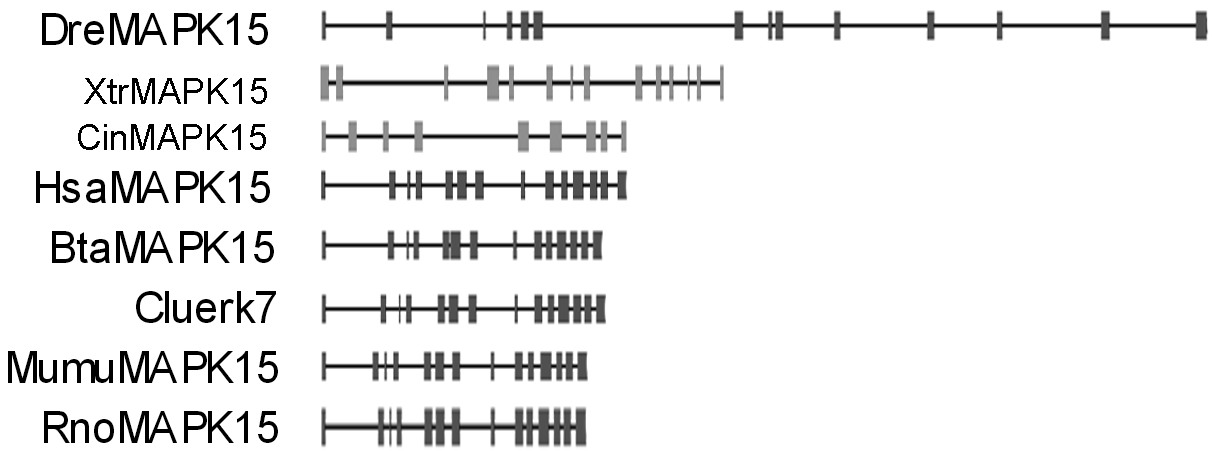


**MAPK15 subfamily**
