# Supplementary material for: Evolutionary History of the Vertebrate Mitogen Activated Protein Kinases Family
Source: PLoS One. 2011 Oct 26;6(10):e26999. doi: 10.1371/journal.pone.0026999 (PMC3202601; doi:10.1371/journal.pone.0026999)
Supplement: Table S2 — Free model test for each MAPK subfamily. (DOC) [file pone.0026999.s008.doc]

**Table S2. Free model test for each MAPK subfamily.**

| **Subfamily** | **dN/dS ^a^**  **one-ratio** |  | **lnL ^b^** | | **2Δl** | **P value** |
| --- | --- | --- | --- | --- | --- | --- |
|  |  | **d.f. ^c^** | **one-ratio** | **free-ratio** |  |  |
| MAPK1 | 0.002 | 18 | -3140.104 | -3130.273 | 9.832 | 0.937 |
| MAPK3 | 0.033 | 20 | -4543.686 | -4510.641 | 33.045 | 0.033 |
| MAPK4 | 0.054 | 22 | -6108.536 | -6062.961 | 45.575 | 0.002 |
| MAPK6 | 0.019 | 28 | -4798.953 | -4778.878 | 20.075 | 0.862 |
| MAPK7 | 0.037 | 24 | -11800.369 | -11744.537 | 55.832 | 0.000 |
| MAPK8 | 0.022 | 18 | -3308.220 | -3288.693 | 19.526 | 0.360 |
| MAPK9 | 0.011 | 30 | -3600.005 | -3581.658 | 18.347 | 0.953 |
| MAPK10 | 0.011 | 16 | -4049.454 | -4016.516 | 32.938 | 0.008 |
| MAPK11 | 0.018 | 20 | -3388.936 | -3378.880 | 10.055 | 0.967 |
| MAPK12 | 0.026 | 28 | -2884.927 | -2869.247 | 15.680 | 0.970 |
| MAPK13 | 0.052 | 18 | -4842.656 | -4819.378 | 23.278 | 0.180 |
| MAPK14 | 0.043 | 24 | -4463.862 | -4393.043 | 70.819 | 0.000 |
| MAPK15 | 0.130 | 8 | -6398.588 | -6370.059 | 28.528 | 0.000 |

^a^The single dN/dS value (average over all sites) for each MAPK subfamily was obtained by M0 model (one-ratio model);

^b^lnL: the log-likelihood difference between the two models; 2Δl: twice the log-likelihood difference between the two models；

^c^d.f. (degrees of freedom): difference in number of parameters between models.

In MAPK subfamilies 3, 4, 7, 10, 14, 15, the free-ratio model are favored (p<0.05), suggesting that there are variable selective pressures.
