# Supplementary material for: Evolutionary History of the Vertebrate Mitogen Activated Protein Kinases Family
Source: PLoS One. 2011 Oct 26;6(10):e26999. doi: 10.1371/journal.pone.0026999 (PMC3202601; doi:10.1371/journal.pone.0026999)

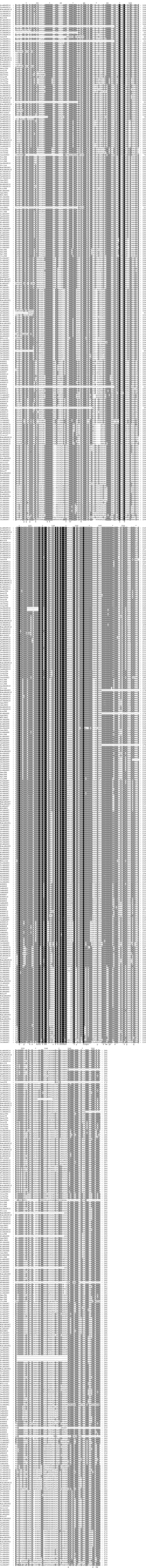

Supplement: Figure S3 — Complete amino acid sequence alignment of MAPKs from vertebrates, tunicates, echinoderm, nematodes, arthropods and plants. This alignment was used to reconstruct the Bayesian phylogenetic tree shown in Figure 2. (PDF) [file pone.0026999.s003.pdf]
